# Analysis of the Impact of the Post-Consumer Film Waste Scenario and the Source of Electricity on the Harmfulness of the Mass Packaging Process

**DOI:** 10.3390/polym16243467

**Published:** 2024-12-12

**Authors:** Patrycja Walichnowska, Weronika Kruszelnicka, Izabela Piasecka, Józef Flizikowski, Andrzej Tomporowski, Adam Mazurkiewicz, José Miguel Martínez Valle, Marek Opielak, Oleh Polishchuk

**Affiliations:** 1 Faculty of Mechanical Engineering, Bydgoszcz University of Science and Technology, al. Prof. S. Kaliskiego 7, 85-796 Bydgoszcz, Poland; izabela.piasecka@pbs.edu.pl (I.P.); jozef.flizikowski@pbs.edu.pl (J.F.); andrzej.tomporowski@pbs.edu.pl (A.T.); adam.mazurkiewicz@pbs.edu.pl (A.M.); 2 Department of Mechanics, Building Leonardo Da Vinci, Campus of Rabanales, University of Córdoba, Cta. Madrid-Cádiz, Km. 396, 14071 Córdoba, Spain; jmvalle@uco.es; 3 Faculty of Transport and Informatic, University of Economics and Innovation in Lublin (WSEI), Projektowa 4, 20-209 Lublin, Poland; m.opielak@pollub.pl; 4 Faculty of Engineering Mechanics, Khmelnytskyi National University, Instytuts’ka Str., 29016 Khmelnytskyi, Ukraine; opolishchuk71@gmail.com

**Keywords:** polyethylene film, disposal scenario, circular economy, technological process, impact on environment

## Abstract

Life cycle analysis (LCA) is a popular tool for determining the environmental impacts of a product in use. The aim of this study is to carry out a life cycle analysis, gate-to-gate, of a mass packaging process using a polyethylene shrinking film with a focus on energy consumption, raw material use and associated emissions, and film post-consumer disposal scenarios. Two different scenarios for the disposal of the shrinking film used in the packaging process were analyzed, namely recycling and landfills. The analysis showed that choosing recycling as the post-consumer management of film waste within the studied system boundaries reduces the negative environmental impact by approximately 17%. The study showed significantly higher environmental benefits in terms of harmfulness to human health for recycling than for landfills. A study of the environmental impact of the mass packaging process depending on the energy source showed that using a renewable source minimizes environmental damage. Three sources of energy options were analyzed, including the country’s energy mix, wind, and solar. The research shows that changing sources to wind power reduces potential damage to human health by 91%, to ecosystems by 89%, and to resources by 92% compared to the country’s energy mix power option. When comparing the results for the renewable energy options, the variant with energy from wind presents lower harm in all three damage categories compared to the solar option.

## 1. Introduction

The modern packaging industry is struggling with many challenges related to reducing the negative impact on the environment. Mass bottle packaging processes are often associated with the extensive use of heat-shrinkable polyethylene film, which is important in protecting and transporting beverage bottles [1,2]. Although film packaging is an effective and cheap solution, its widespread use leads to serious waste management problems, which have a significant impact on the environment. After fulfilling its function, i.e., delivering the product to the recipient, the heat-shrinkable film becomes waste [3]. If not properly managed, it contributes to large volumes of residue that are difficult to dispose of, which has a negative impact on the environment [4,5,6]. In Poland, according to Plastics Europe in 2022, recycling accounted for 21% in the management of plastic waste, meaning that just over one fifth of all plastic waste was recycled to reuse raw materials [7]. Energy recovery, which accounted for 35%, indicates the use of technology to convert plastic waste into energy, which is beneficial in terms of reducing the amount of waste going to landfills, but at the same time limits the possibility of reusing raw materials. Landfilling accounted for the largest share of plastic waste, at 44%. This indicates the need to implement new technologies and measures to raise people’s awareness of the environmental impact of this type of management [7]. Due to the above, the aim of this work was to determine the potential environmental impact of the bottle packaging process using heat-shrinkable film in two variants of the post-consumer waste management scenario in the form of film—recycling and storage. The presented analysis is a continuation of research in the field of the mass bottle packaging process. So far, the authors have focused on the possibilities of changing the film used in the process to a film from recycling. The analyses carried out have shown that the film with a 50% addition of recyclates can replace the traditional polyethylene film [8] while also reducing the harmfulness of the process. In the case of considering the 100% recyclable film, it has been shown, among other things, that its tensile strength is about 45% lower than that of the traditional film; therefore, it is necessary to further introduce changes in the film structure to obtain functional properties similar to those of the traditional film [9]. In this article, research was conducted on the impact of the post-consumer management scenario of the film on the harmfulness of the process, in which the film with 50% recyclates was used. Additionally, an analysis of the harmfulness scenarios of the packaging process was conducted depending on the power source, including renewable sources (photovoltaic installation and wind installation) without considering post-consumer management.

For years, a large amount of plastic has been produced, which is used, among other things, to produce packaging that is quickly discarded, which if not properly managed, becomes a big problem for the environment [10]. The film-blowing extrusion process is the most used industrial technique to produce heat-shrinkable films, as it allows for achieving the desired properties without additional costs compared to, for example, double-bubble technology. In the blown-film extrusion process, polymer macromolecules can be oriented in two directions, the machine direction (MD) and transverse direction (TD). Based on the degree of orientation in the MD and TD, heat-shrinkable films can be divided into two main groups, comprising mono-oriented films, which are characterized by shrinkage in the MD at the level of 60–80% and in the TD at the level of 10–20%, and bi-oriented films, where the shrinkage is 50–60% in the MD and 30–40% in the TD, respectively. Films can also be divided according to their thickness, with thin heat-shrinkable films (with a thickness of up to 50 μm) and thicker ones (with a thickness of 50 to 150 μm) [11]. Potential opportunities to improve energy efficiency in extrusion and shrink processes include the use of modern high-efficiency heating systems, the optimization of process parameters such as temperature, extrusion speed, and pressure, and the use of advanced technologies such as ultrasound-assisted extrusion. In addition, waste reduction can be achieved through internal recycling, where waste from production is recycled back into the extrusion process, and the implementation of real-time quality monitoring systems is used to minimize defects and rejects. The shift from multilayer materials to monomaterials can also contribute to easier recycling and reduce the amount of difficult-to-process waste [12,13].

The chemical structure of polymers, consisting of long hydrocarbon chains, is exceptionally resistant to natural degradation processes, which leads to their long-term retention in the environment. Polyethylene films are characterized by good mechanical strength, which makes them often used in packaging processes. Unfortunately, this feature also makes it difficult to decompose them at the end of their life cycle, increasing the amount of plastic waste [14]. The degradation characteristics of this type of film indicate its low susceptibility to decomposition under the influence of biological and chemical factors, which results in the accumulation of microplastics in ecosystems and a potential negative impact on living organisms. All these properties emphasize the need for appropriate management of PE waste and the development of recycling technologies and alternative materials with better biodegradability. A way to minimize the amount of polymer waste is recycling, which involves collecting plastic waste and reprocessing the material into useful products. Before processing, most plastics are sorted, which allows for an efficient and appropriate processing of different types of plastics [15].

The recovery of plastics can be carried out through three processes, which are mechanical recycling, raw material recycling, and energy recovery [16]. Mechanical recycling involves processing polymer waste in a process that involves segregation, shredding, washing, and melting, which allows the material to be reused without changing its chemical structure. The waste is shredded into smaller fragments, cleaned of contaminants, and processed into granulation that can be used as a raw material in further production. The properties of mechanically recycled polymers do not remain the same due to degradation caused by heat, mechanical stress, oxidation, and ultraviolet radiation during reprocessing and use. Raw material recycling, which is also called chemical recycling, involves converting the polymer chain into shorter hydrocarbon components that can be used as a source for rebuilding new polymers [17]. Another method is energy recovery, which involves burning waste materials to recover energy. This technology helps reduce the amount of waste in landfills, but its use requires appropriate filtration systems to minimize the emission of harmful substances into the atmosphere [18].

The tensile strength and elasticity of the heat-shrinkable film have a significant impact on environmental efficiency, as they determine the amount of raw material needed for production and affect the durability of the resulting seal during transport. Tensile strength allows for the use of thinner films without losing their functionality, which leads to reduced material consumption and reduced waste generated during production and after use. Flexibility, in turn, affects the film’s ability to adapt to the shape of products, which allows for a more efficient use of packaging and a reduction in losses resulting from damage during transport or storage. Good mechanical properties of LDPE film can also increase its durability and resistance to tearing, which reduces the need for multi-layer structures or additional protective materials that make recycling difficult [8,19].

The available literature contains many examples of conducted environmental analyses considering the post-consumer management of plastic waste. Aryan et al. [20] assessed the environmental impact of different scenarios of plastic waste management for a selected area of the city of Dhanbad in India using life cycle analysis (LCA). The authors showed that the scenario in which recycled products were used and then recycled is characterized by the lowest negative impact on the environment. Dong et al. [21] examined the potential environmental impact of five post-consumer management scenarios of films for agricultural mulching using LCA. The authors showed that the use of biodegradable film reduces CO_2_ emissions by about 23%, and recycling polyethylene film increases the harmfulness in terms of toxicity while reducing the negative impact in terms of resource damage. Ahamed et al. [22] conducted an environmental analysis for plastic packaging with RFIDs, considering different end-of-life scenarios. The study showed that the variant using mechanical recycling of plastic with energy conversion from waste and the chemical recycling of RFIDs is characterized by lower harm in all impact categories except stratospheric ozone depletion potential. The sensitivity analysis showed that chemical recycling may be potentially the best solution due to the supply of high-quality recyclates. Piasecka et al. [23] conducted an eco-energy analysis of the life cycle of materials and components of a 1 MW photovoltaic power plant based on LCA. The authors showed that the use of recycling processes would reduce the potential negative impact on the environment throughout the life cycle. Piotrowska and Piasecka [24] conducted a similar analysis for eight types of waste generated during the production of wind turbine blades. The analysis concerned determining the impact on three categories of damage, i.e., human health, ecosystems, and resources. The greatest potentially negative impact was shown for resin disks. The authors indicated that the use of recycling processes would reduce the negative impact throughout the entire life cycle of the objects studied. The circular economy model for plastics is a real alternative to the current linear system, in which plastics are produced, used, and then disposed of, which is why it is such a common topic in current research [25,26,27,28,29].

The main objective of this article is to compare the environmental impact of the mass packaging process depending on the method of waste management in the form of heat-shrinkable film and the source of electricity powering the process in two different limits of the studied system. Such an assessment of harmfulness is consistent with the current trends in the search for an effective and sustainable use of plastics. The results obtained in the article can be used to design modern solutions in the field of plastic waste management and the optimization of the mass packaging process. The analysis conducted will help to understand how the post-consumer management of film waste can reduce its negative impact on the environment and can lead to optimization of the mass packaging process of bottles. Leveraging this research enables the introduction of waste-minimizing technologies and practices, effectively driving down production costs. In the available literature, there is a lack of studies that refer to conditions in Poland. This article aims to determine the environmental impact of the research object studied from a local point of view.

## 2. Materials and Methods

The article is a continuation of research on the analyzed technical system. Referring to the results from previous articles [8,9], the analysis assumed the use of film with a 50% addition of recyclates (50RF) for packaging bottles. The tests of functional properties presented in previous articles showed that the 50RF film had a higher linear free shrinkage of around 10%, tensile strength in the LD direction was 10% lower, and tear strength was about 60% higher than for traditional heat-shrinkable film. The obtained results allowed us to state that the tested batch of 50RF film can replace traditional film (without added recyclates) in the mass process of packaging bottles. The analysis of the harmfulness of the process depending on the film used showed that changing the type of film to film with added recyclates results in an almost 70% reduction in the negative impact of the process on health and ecosystems and by 85% on resources. Continuing the considerations of the harmfulness of the mass packaging process, it was decided to conduct further investigations on the impact of post-consumer management of the film used in the studied process. There are several methods of managing plastic waste, including landfills and mechanical or chemical recycling. Recently, it has been shown that the recycling process is a more ecological alternative to processing polymer waste compared to old methods [30,31].

**Figure 1:**
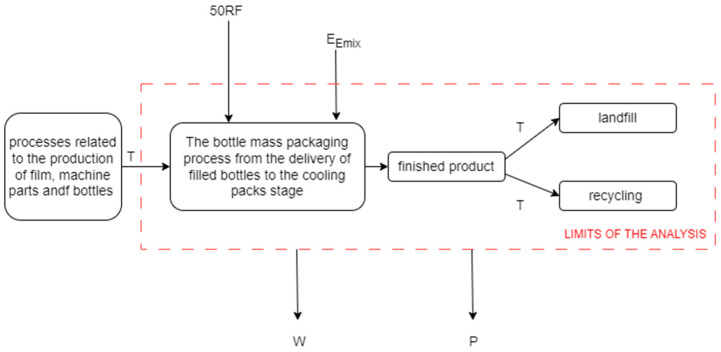
A diagram of the process of the mass packaging of bottles including input and output data. E_Emix_—electricity in the process variants; 50RF—used recycled film in all variants of the process; P—pollution in the form of greenhouse gas emissions (CO_2_, SO_2_, SF_6_, N_2_O); W—waste in the form of unusable packs; T—transportation (own study).

To determine the impact on the environment, a gate-to-gate life cycle assessment of the mass packaging process was carried out. This process of the mass packaging of bottles was carried out on a packaging machine equipped with feeding conveyors at the input and output, which are responsible for transferring the goods for wrapping and automatic unloading of the already packaged product. The machine carries out the process of the mass packaging of bottles, which aims to combine unit elements (bottles with beverages) into integrated packages by wrapping them in film and then shrinking it using hot air in a heating oven. The case described in the article was based on a traditional packaging process powered by energy from the country’s energy mix in the food industry, the final product of which is a packed pack of beverage bottles. Five stages were specified in the tested technological process, comprising supplying bottles, bottle formatting, wrapping a group of bottles with film, shrinking the film around a group of bottles, and cooling packs falling within the limits of the tested system (Figure 1). The analysis was performed for two different post-consumer scenarios, namely landfills and recycling.

Life cycle assessment programs such as GaBi, MiLCA and OpenLCA include SimaPro 9.6 software, which is based on the estimation of environmental impacts using databases, e.g., Ecoinvent, and advanced calculation methods (e.g., ReCiPe 2016), and it is based on assumptions similar to the philosophy of G. Taguchi, where the place of losses is exchanged for damage caused to the environment during the life cycle of the tested object [32]. The damage assessment is carried out based on estimating the loads assigned to the appropriate category of impacts. To reduce the number of impacts, the environment has been defined as a set of biological, physical, and chemical parameters constituting necessary conditions for the functioning of people and nature, which are directly or indirectly influenced by human activity. The assessment can be carried out provided that an appropriate research method is selected for the purpose [33,34].

The analysis used data from the Ecoinvent v3 database, which includes electricity from the Polish national energy mix, its import, and its conversion to low voltage, along with the losses that occurred. The environmental analysis of the tested process was carried out using SimaPro 9.3 software with the Ecoinvent v3 database. SimaPro uses mathematical models for product life cycle assessment (LCA) in accordance with ISO 14040 and ISO 14044 standards [34,35]. The tool uses mass and energy balance equations that analyze the quantitative flows of inputs and outputs in each process that makes up the system [36]. For example, for a production process, the amount of raw materials needed to make a product, such as oil in the case of plastic, and the emissions generated in the process, such as carbon dioxide, are calculated. These data are then processed by environmental impact assessment algorithms that use the combination of emission flows with the appropriate impact categories [37]. In the LCA conducted in accordance with the standards [34,35], the ReCiPe 2016 method was used, which is a valued tool for assessing the harmfulness of, among others, technological processes. In this method, there is a division into midpoint categories, which concern the assessment of direct ecological effects, and endpoint categories, which focus on the final effects of the tested object for three categories of damage, i.e., ecosystems, human health, and resources. ReCiPe 2016 also allows for the consideration of different time perspectives, such as short-term, medium-term, and long-term perspectives, which makes it possible to differentiate the results depending on the adopted analysis assumptions [38,39,40]. Data from 2023 from an actual object—a company involved in the mass packaging process of bottles—were used to conduct the research. From the analysis of data from the company over the last few years, it was assumed that deficiencies in the form of defective packaging constitute 1% of the total annual production. To provide a reference for normalizing, the data input and output of the research technical system were adopted with a functional unit as 1000 packs. The data presented in Table 1 were used to conduct the analysis. Due to a limited amount of information, it was assumed that the use of the film takes place within one province, which allowed us to assume the distances between the factory, the user, and the recipient of the film waste as 90 km traveled by a truck, which is characterized by fuel consumption at the level of 27.6 l/100 km at a driving speed of up to 120 km/h. To determine the fuel consumption expressed in l/100 km to fuel consumption in l/tkm, it was necessary to consider the mass of the load transported by the vehicle. In the SimaPro program, the emission inventory for the transportation stage was modeled using a truck with a load capacity of 24.7 tons.

The study also included a comparison of mass packaging process scenarios depending on the energy source used in the process, as shown in Table 2. In the scenario analysis, only the source of energy was changed. Studies were conducted for the following scenarios:

- Scenario I: electricity from the country’s electrical mix.
- Scenario II: electricity from a wind farm.
- Scenario III: electricity from a photovoltaic farm (PV farm).

**Figure 2:**
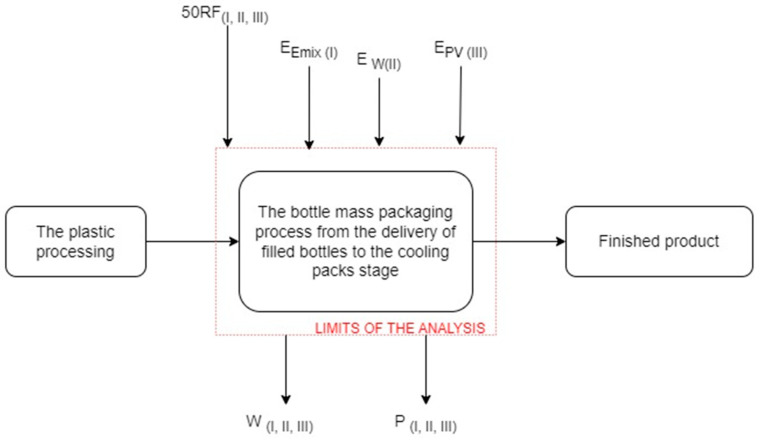
A diagram of the process of the mass packaging of bottles including input and output data. E_Emix(I)_—electricity in process variant I; 50RF—used recycled film in all variants of the process; E_W(II)_—electricity in process variant II; E_PV(III)_—electricity in process variant III; P_(I, II, III)_—pollution in the form of greenhouse gas emissions (CO_2_, SO_2_, SF_6_, N_2_O); W_(I, II, III)_—waste in the form of unusable packs (own study).

This part does not include the post-consumer management of film waste. The boundaries of the studied system included the mass packaging process from the delivery of filled bottles to the cooling packs stage (Figure 2).

In the first scenario of variant B, the energy supplying the process in question comes from the country’s energy mix, which was dominated by hard coal (46.92%) and brown coal (19.60%). When it comes to green energy, wind farms contributed 19.97% to energy production (Table 2). The second variant assumed powering the machines with energy produced from a wind farm and the third from a photovoltaic farm. In this study, the data from Ecoinvent databases refers to the geographical area of the European Union 28 (EU-28). In the analysis, unit processes connected with the raw material acquisition and production processes of shrinkable film, as well the averaged unitary process of electricity production from the country’s energy mix and electricity production from wind power plants and PV farms were applied from the Ecoinvent database to estimate the environmental impact. It should be noted that in the Ecoinvent database, the averaged data of impact for electricity production from solar and wind installations include the whole life cycle environmental impact for those installations; so, not only is the phase of electricity generation included but the raw material acquisition, production stage, and end-of-life treatment of components are also considered. All studies and data refer to a period of the last several years.

## 3. Results

### 3.1. The Analysis of Harmfulness Due to a Disposal Scenario

The conducted environmental analysis comparing the impact of the method of managing film waste on the harmfulness of the mass packaging process of bottles showed that recycling reduces the negative potential impact in the category of damage to human health and resources. Table 3 presents the results of characterizing the environmental consequences occurring in two compared variants of the process of packaging bottles in heat-shrinkable film. In the category of damage to human health, the introduction of recycling reduced the potential damage by about 18% compared to the landfill scenario. Only the damage within global warming was characterized by a potentially higher value of impacts in this category. Analyzing the results obtained for the resource damage category, a negative impact of less than 1% is noticeable for the variant with recycling than for the variant with landfills.

Based on the analysis carried out, it was found that the impact categories with the potentially highest level of negative impact on the environment in both variants tested in all damage categories are global warming, human health (VA: 1.04 × 10^−4^ DALY and VB: 1.05 × 10^−4^ DALY), human non-carcinogenic toxicity (VA: 1.07 × 10^−4^ DALY and VB: 5.37 × 10^−5^ DALY), freshwater eutrophication (VA: 8.80 × 10^−8^ species.yr and VB: 1.51 × 10^−7^ species.yr), and fossil resource scarcity (VA: USD 3.52 and VB: USD 3.47).

**Figure 3:**
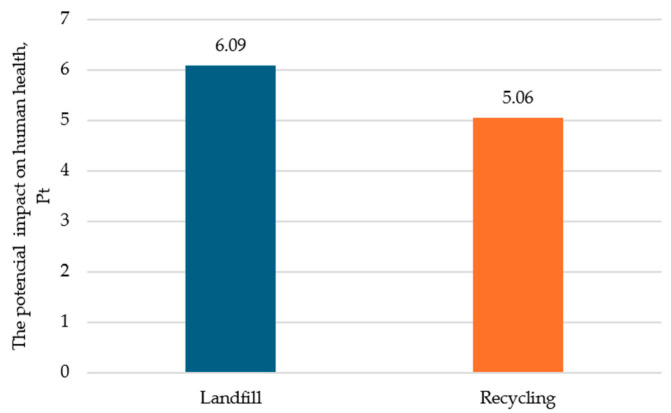
The impact of the mass bottle packaging process in the damage category of human health, Pt (own study).

The main objective of the article was to indicate how the type of post-consumer management of film waste affects the harmfulness of the mass packaging process of bottles. As a result of this analysis, the impact of individual variants in three categories of damage expressed by environmental points was determined. For the category of damage to human health, the variant of the tested research object with recycling was characterized by a lower potential negative impact by 1.03 Pt (Figure 3).

**Figure 4:**
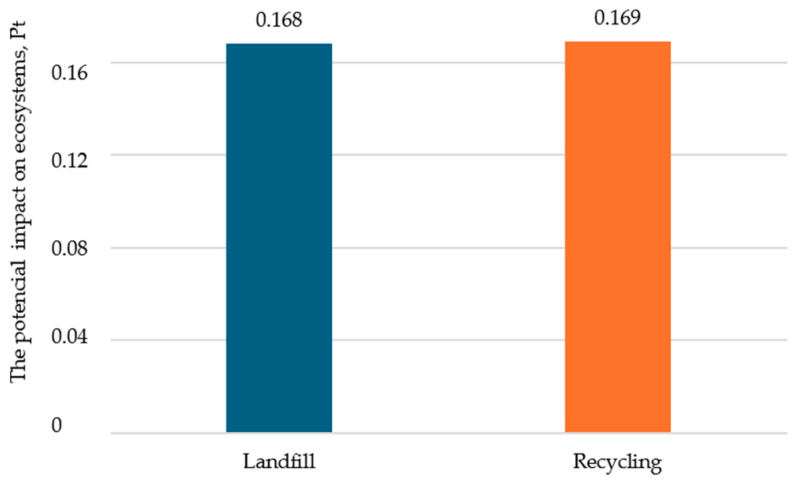
The impact of the mass bottle packaging process in the damage category of ecosystems, Pt (own study).

For the category of damage to ecosystems, a potentially greater negative impact is noticeable for the disposal scenario variant in the form of recycling (Figure 4). However, it is important to note that this difference amounts to only 0.001 Pt.

**Figure 5:**
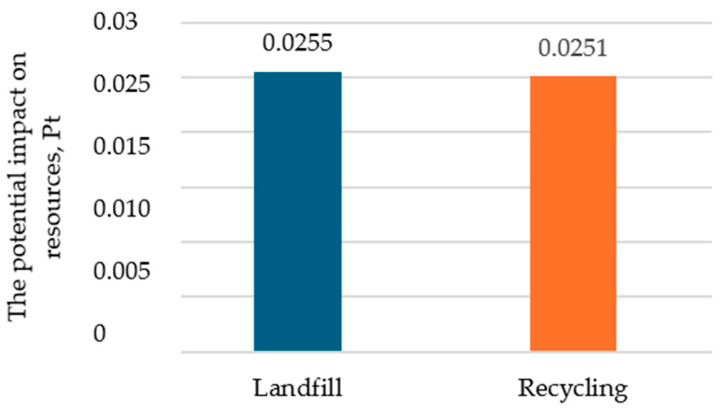
The impact of the mass bottle packaging process in the damage category of resources, Pt (own study).

For the damage category “Resources”, the potential environmental impact is reduced in scenarios involving recycling. The second variant is characterized by an impact in this damage category at the level of 0.0251 Pt and is about 2% smaller than for the landfill variant (Figure 5).

### 3.2. The Analysis of Harmfulness Due to the Source of Electrical Energy

In all analyzed cases, changing the energy source reduced the harmfulness of the tested process. In the category of damage to resources, changing the source of electricity results in a significant reduction in the value of revenues for the two unconventional variants of the examined process (Table 4). In the category of damage to human health, the first variant of the process is characterized by an impact of 0.000174 DALY. Much lower values determined for the proposed two unconventional process variants for which the impact value in terms of damage to human health in the discussed case is 0.0000163 DALY for the second variant and 0.0000191 DALY for the third variant, respectively.

**Figure 6:**
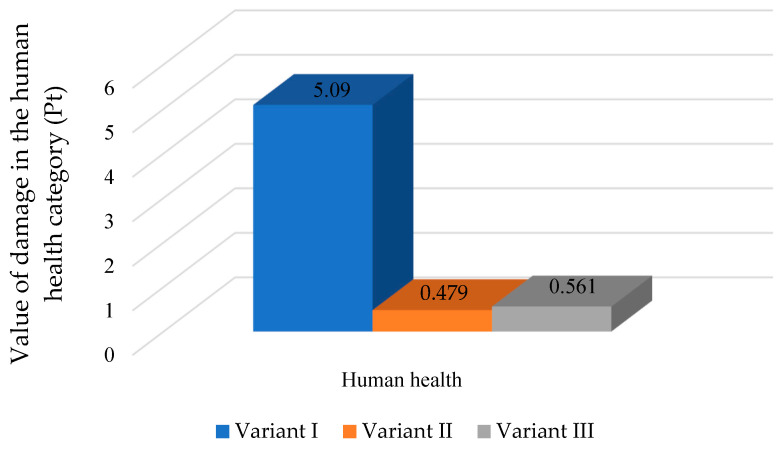
The impact of the mass bottle packaging process on three variants depending on the source of electricity in the damage category of human health (Pt) (own study).

In the analyzed variants of the mass bottle packaging process, changing the energy source to renewable sources reduces the harmfulness of the tested process. As can be seen in Figure 6, in the category of damage to human health, changing the source of electricity reduces the impacts in this category. There is a slight advantage for variant II, where the impact value in terms of damage to human health is characterized by the lowest value (0.479 Pt).

**Figure 7:**
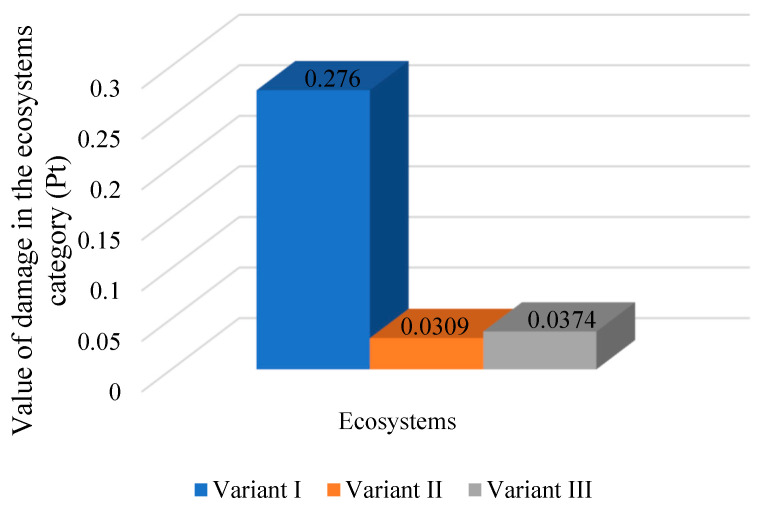
The impact of the mass bottle packaging process on three variants depending on the source of electricity in the damage category of ecosystems (Pt) (own study).

In the category of damage to ecosystems (Figure 7), there is also a noticeable reduction in the value of environmental consequences when changing the energy source to non-conventional ones. In this damage category, the lowest damage values were determined for the second variant (0.0309 Pt). In the variant powered by energy from a photovoltaic installation, the impact is 0.0374 Pt. However, the highest impact in the ecosystem damage category, amounting to 0.276 Pt, is characteristic of the traditional variant of the process powered by the country’s energy mix.

**Figure 8:**
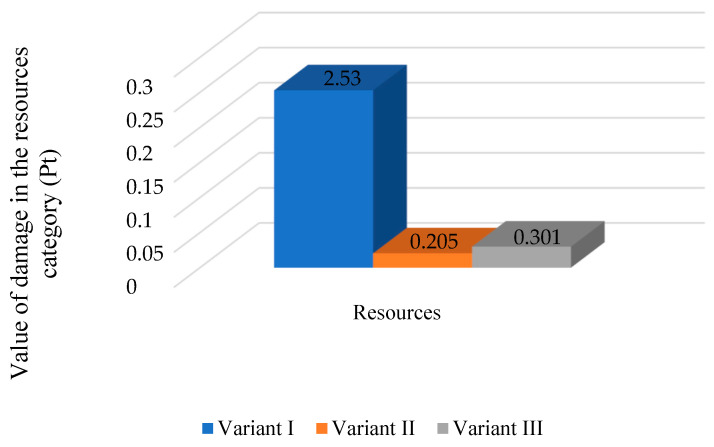
The impact of the mass bottle packaging process on three variants depending on the source of electricity in the damage category of resources (Pt) (own study).

Comparing the results of the impact on resources (Figure 8), changing the energy source to renewable ones, similarly to the previous damage categories, reduces the impact of the mass bottle packaging process. For the first variant, the impact in this category was set at 2.53 Pt. However, changing the power source to wind energy reduces the damage to the analyzed process by 0.205 Pt. Changing the power supply to energy from a photovoltaic installation also resulted in a reduction in the damage value (0.301 Pt) compared to the first variant.

## 4. Discussion

A significant challenge associated with the plastics industry is the continually increasing volume of non-degradable waste. Plastic materials are notable for their ease of production and low manufacturing costs, factors that contribute to their extensive use, including in the food industry [15,41]. Recycling is an important element in implementing a circular economy. However, the creation of multilayer films from different polymers makes recycling problematic. Another significant problem in recycling films is the limitations occurring at the sorting stage and the quality of the films received, which are often contaminated. Currently, only commercial and industrial films are recovered through open mechanical recycling [15]. The research conducted in the article showed a lower potential negative impact on the environment of recycling for the category of damage to human health by about 18% and by about 2% for resource depletion. The process of storing the film in landfills leads to long-term decomposition of the material, during which harmful substances and methane, which contribute to global warming, are released. In contrast, recycling allows for the recovery of the raw material and its reuse, which reduces the need for the production of new granulates, reduces CO_2_ emissions, and also limits the demand for natural resources [42,43,44]. Film waste management is a critical component of a circular economy. Improper processing of this waste presents significant environmental risks. While recycling generally shows lower values for negative environmental impact, it is associated with high energy demands that, in the absence of renewable energy sources, impose substantial environmental burdens. Therefore, ongoing research should focus on evaluating the potential environmental impact of transitioning film waste processing power supplies to renewable energy sources.

Technological processes are an integral aspect of the development of economies in all countries around the world. As the demand for a given product, including beverage bottles packed in film, increases, the demand for electricity also increases. Due to this situation, it is considered necessary to search for and analyze solutions that will simultaneously provide the appropriate amount of energy to power processes, including the process of the mass packaging of bottles in thermo-shrinkable film, and at the same time reduce the harmfulness associated with them [45,46].

The environmental assessment results obtained as part of simulation studies are largely related to the consumption of electricity from the country’s energy mix, which, for example, in Poland, is approximately 47% from hard coal and approximately 20% from lignite. The rest of the components are energy produced from natural gas, industrial gas, and renewable energy power plants. The analysis carried out in this work of changing the power source of machines to energy coming exclusively from wind farms and photovoltaic plants showed a reduction in the negative impact of the tested mass packaging process in all categories of damage. In the three categories of damage, the lowest potential impact indicators were observed in variant II of the mass bottle packaging process, which was powered by energy from a wind farm. The lower values of environmental impact in the case of powering the process by the wind power plant than by the PV power plant is caused by the differences in the impacts connected with the raw material acquisition and end-of life treatment of these two alternative energy sources [47]. As was mentioned, the data for energy production from renewables in Ecoinvent are averaged, including all life cycle stages. For PV and wind farms, the impact during the use stage is marginal, but those connected with raw material acquisition, production, and end-of-life are most dominant [47,48,49]. As was shown [47], in the case of PV installation, the most critical aspect is the use of rare minerals, such as selenium, to produce modules. These rare minerals will eventually be exhausted if solar panel manufacturers continue to extract them. Moreover, the recycling technologies of PV modules are limited, so not all of the materials and energy potentials can be recovered [50,51]. In the case of wind power plants, the most harmful are non-recyclable plastic composite materials used in the blades of a wind turbine [47], but the high percentage (even up to 90% [47,52]) of the materials and energy in the wind power plant life cycle can be recovered, which reduces the impacts. The usage of rare earth materials with high environmental impact and a low percentage of recycling of crystalline modules are then the main reasons why the impacts from powering the packing process are the lowest for variant II in the presented study. The results obtained are consistent with those of other studies regarding the carbon footprint. In Hamed et al.’s work [49], it was concluded that the amount of greenhouse gas emissions produced depends on the power source. For coal, it is on average approx. 950 g CO_2_/kWh, for PV installations containing monocrystalline modules, 50 g CO_2_/kWh, and for wind energy, around 20 g CO_2_/kWh [49]. The results indicate slightly higher impact values for the variant with solar energy compared to the second variant. However, this solution still reduces the harmfulness of the process and is an alternative to the traditional method of supplying the process with energy from the country’s energy mix. To reduce the harmfulness of the mass packaging process, it is reasonable to increase the share of renewable energy in the total energy consumption, in particular, energy obtained from wind. Therefore, in terms of environmental impact, the company could invest in its own power source. Of course, it would be necessary to consider the aspects of the economic profitability of such an action, i.e., check whether the savings in terms of reducing electricity bills and CO_2_ emissions would exceed the investment costs in a relatively short time. The result, indicating a higher potential impact of variant I (energy from the country’s energy mix) on the resource damage category expressed in dollars, is justified due to the non-renewable nature of the country’s energy mix based on coal and the associated increasing costs of future exploitation resulting from its depletion. The increased energy and financial input required to extract hard-to-reach resources contributes to a higher excess cost, which is included in the damage category [53]. In the ReCiPe 2016 methodology used in life cycle analysis (LCA), the impact on resources quantifies the excess cost of future resource extraction resulting from current consumption, focusing on the depletion of mineral and fossil resources. The excess cost reflects the additional economic burden that society will bear as resources become increasingly scarce and expensive to exploit [54]. The unit of resource scarcity is dollars (USD), which represent the additional costs associated with the future exploitation of mineral and fossil resources.

For the final generalizing conclusions and recommendations, the limitations of this study should be considered. The results presented were based on specific production lines with specific operating parameters and production efficiency for specific regional boundaries. It should be noted that for other installations with higher or lower energy efficiency and productivity, the results may differ. In this study, the regional border of the analysis was the EU, and the Polish energy mix was considered. For the regions and energy mixes with a higher share of non-renewable energy, the reduction in environmental impacts by changing the sources of energy to renewable ones will be probably greater than in this study. Moreover, data on obtaining energy from individual sources were taken from the Ecoinvent database, for example, for a specific type of PV installation. Even by changing the type of selected PV modules or wind turbines, the environmental impacts would be different [49]. One of the limitations is also the failure to consider the post-consumer management of finished products (packs). It is challenging to build a specific scenario and obtain the data for all processes involved in the end-of-life of used packs, such as collecting, sorting, washing, recycling, or disposal, as there are large differences in the distances to travel for the collection, materials, and energy usage of processing from site-to-site.

## 5. Conclusions

The future of film packaging used in bottle transport requires the implementation of modern materials (e.g., biodegradable) and a circular economy in the post-consumer management of film waste. This approach involves designing packaging that is readily recyclable or compostable, alongside developing more sustainable systems for the collection and processing of used packaging. Such measures have the potential to substantially reduce the environmental impact associated with film waste. The limited lifecycle analysis conducted here indicates the following:

- The use of recycling as a method of a post-consumer scenario of film waste from the process of the mass packaging of bottles reduces the potential impact on the environment within the specified limits of the tested system;
- The use of landfills as a disposal scenario in the adopted functional unit causes approx. 17% greater environmental burden in all three tested damage categories;
- The tested recycling variant shows significantly lower damage in terms of the impact on human health (human health) and the consumption of natural resources (resources) compared to the landfill option. The recycling process, through the reprocessing of materials, reduces the emission of harmful substances and pollutants that can be released into the air, soil, and groundwater during waste storage;
- Recycling plastic waste can be considered a sustainable waste management practice. The results obtained indicate that further considerations in this area should be continued to minimize the potential impact within ecosystems;
- To obtain a more complete picture of the impact on the environment, it is planned to extend the analysis in the next stage of the research to include specific recycling methods used for heat-shrinkable films, such as mechanical, chemical, or thermal recycling. Each of these types of recycling differs both in terms of processing technology and the impact on CO_2_ emissions and energy consumption;
- Continuing research around plastics recycling and implementing the results of these analyses in practice will enable the creation of more sustainable solutions that will not only reduce the impact on the environment but also support the construction of a circular economy.

The environmental analysis of the mass bottle packaging process, carried out in the next step, depending on the type of energy source powering the process, showed the following:

- Conducted research has shown that powering the process with energy obtained from wind is characterized by the least harm among the tested process variants in all three categories of damage;
- Changing the power source in the discussed process to energy obtained from wind reduces the potential damage to human health by about 91%, to ecosystems by about 89%, and in resources by about 92%;
- The lower environmental impact values of variant II compared to variant III result from differences in the impacts related to the extraction of raw materials and the final processing of these two alternative energy sources;
- The analysis carried out allowed for the illustration of potential changes in the impact on the surroundings in the event of a change in the power source. It should be emphasized, however, that such a change requires large financial outlays from the company and the adjustment of production time to the highest daily energy yields from individual renewable sources to use the energy produced as effectively as possible;
- The use of domestic renewable sources will reduce dependence on fossil fuel imports, which will increase the country’s energy security.

Based on the research conducted, it should be stated that both considerations in the post-consumer management of film waste and in the scope of energy used during the implementation of the process in question are important for introducing changes that will contribute to reducing the harmfulness of the process and its optimization. Recycling film waste provides a great chance to limit the negative effects of the process of the mass packaging of bottles, but it is still a challenge that requires work in the field of appropriate waste management. Changing the energy source to unconventional sources contributes to reducing the harmfulness of the process but requires entrepreneurs to implement a new energy management system, including investments in the construction of local renewable energy source installations.

## Figures and Tables

**Table 1:** Inventory data in two tested variants (own study).

Parameter	Variant A	Variant B
post-consumer scenario	Landfill	Recycling
amount of used electrical energy, kWh	67.81	68.81
amount of used 50RF, kg	31.57	31.57
fuel consumption, l/tkm	0.010	0.010

**Table 2:** Inventory data on energy and film consumption for a scenario analysis (own study).

Parameter	Variant I	Variant II	Variant III
Electrical energy, kWh	67.81 (country electricity mix)	67.81 (wind farm)	67.81 (PV farm)
50RF film, kg	36.40		

**Table 3:** The impact of the mass bottle packaging process in two variants depending on the disposal scenario (own study).

Impact Category	Unit	Variant A (VA)	Variant B (VB)
Global warming (human health)	DALY	1.04 × 10^−4^	1.05 × 10^−4^
Stratospheric ozone depletion	DALY	1.24 × 10^−8^	1.20 × 10^−8^
Ionizing radiation	DALY	4.95 × 10^−8^	4.77 × 10^−8^
Ozone formation (human health)	DALY	1.72 × 10^−7^	1.72 × 10^−7^
Fine particulate matter formation	DALY	8.64 × 10^−5^	8.60 × 10^−5^
Human carcinogenic toxicity	DALY	5.88 × 10^−5^	5.78 × 10^−5^
Human non-carcinogenic toxicity	DALY	1.07 × 10^−4^	5.37 × 10^−5^
Water consumption, human health	DALY	9.45 × 10^−6^	4.99 × 10^−7^
Global warming, terrestrial ecosystems	species.yr	3.13 × 10^−7^	3.17 × 10^−7^
Global warming, freshwater ecosystems	species.yr	8.54 × 10^−12^	8.67 × 10^−12^
Ozone formation	species.yr	2.49 × 10^−8^	2.50 × 10^−8^
Terrestrial acidification	species.yr	8.15 × 10^−8^	8.12 × 10^−8^
Freshwater eutrophication	species.yr	8.80 × 10^−8^	1.51 × 10^−7^
Marine eutrophication	species.yr	6.06 × 10^−11^	2.10 × 10^−11^
Terrestrial ecotoxicity	species.yr	7.81 × 10^−9^	8.64 × 10^−9^
Freshwater ecotoxicity	species.yr	1.45 × 10^−8^	7.39 × 10^−9^
Marine ecotoxicity	species.yr	2.94 × 10^−9^	1.57 × 10^−9^
Land use	species.yr	2.59 × 10^−8^	2.52 × 10^−8^
Water consumption, terrestrial ecosystems	species.yr	6.24 × 10^−8^	7.87 × 10^−9^
Water consumption, aquatic ecosystems	species.yr	2.62 × 10^−12^	1.89 × 10^−13^
Mineral resource scarcity	USD	0.05	0.05
Fossil resource scarcity	USD	3.52	3.47

**Table 4:** The impact of the mass bottle packaging process in three variants depending on the source of electricity in three different categories of damage (own study).

Damage Category	Unit	Variant I	Variant II	Variant III
Human health	DALY	1.74 × 10^−4^	1.63 × 10^−5^	1.91 × 10^−5^
Ecosystems	species.yr	6.33 × 10^−7^	7.1 × 10^−8^	8.58 × 10^−8^
Resources	USD	3.1	0.251	0.369

## Data Availability

The raw data supporting the conclusions of this article will be made available by the authors upon request.
